# Bone Formation in a Rat Tibial Defect Model Using Carboxymethyl Cellulose/BioC/Bone Morphogenic Protein-2 Hybrid Materials

**DOI:** 10.1155/2014/230152

**Published:** 2014-04-06

**Authors:** Sang-Heon Song, Young-Pil Yun, Hak-Jun Kim, Kyeongsoon Park, Sung Eun Kim, Hae-Ryong Song

**Affiliations:** ^1^Department of Orthopedic Surgery, Myongji Hospital, 55 Hwasu-ro 14 Beon-gil, Deokyang-gu, Goyang 412-826, Republic of Korea; ^2^Department of Orthopedic Surgery and Rare Diseases Institute, Korea University Medical College, Guro Hospital, No. 80, Guro-dong, Guro-gu, Seoul 152-703, Republic of Korea; ^3^Division of Bioimaging, Chuncheon Center, Korea Basic Science Institute, 192-1 Hyoja 2-Dong, Chuncheon, Gangwon-do 200-701, Republic of Korea

## Abstract

The objective of this study was to assess whether carboxymethyl cellulose- (CMC-) based hydrogel containing BioC (biphasic calcium phosphate (BCP); tricalcium phosphate (TCP) : hydroxyapatite (Hap) = 70 : 30) and bone morphogenic protein-2 (BMP-2) led to greater bone formation than CMC-based hydrogel containing BioC without BMP-2. In order to demonstrate bone formation at 4 and 8 weeks, plain radiographs, microcomputed tomography (micro-CT) evaluation, and histological studies were performed after implantation of all hybrid materials on an 8 mm defect of the right tibia in rats. The plain radiographs and micro-CT analyses revealed that CMC/BioC/BMP-2 (0.5 mg) led to much greater mineralization at 4 and 8 weeks than did CMC/BioC or CMC/Bio/BMP-2 (0.1 mg). Likewise, bone formation and bone remodeling studies revealed that CMC/BioC/BMP-2 (0.5 mg) led to a significantly greater amount of bone formation and bone remodeling at 4 and 8 weeks than did CMC/BioC or CMC/BioC/BMP-2 (0.1 mg). Histological studies revealed that mineralized bone tissue was present around the whole circumference of the defect site with CMC/BioC/BMP-2 (0.5 mg) but not with CMC/BioC or CMC/BioC/BMP-2 (0.1 mg) at 4 and 8 weeks. These results suggest that CMC/BioC/BMP-2 hybrid materials induced greater bone formation than CMC/BioC hybrid materials. Thus, CMC/BioC/BMP-2 hybrid materials may be used as an injectable substrate to regenerate bone defects.

## 1. Introduction

More than a million people in the United States and other countries require bone graft to regenerate bone defects caused by fracture, trauma, or tumor resection [1, 2]. Autograft has been used widely as bone graft due to its excellent osteoinductive and osteoconductive properties [2]. Allograft has also been used as a bone substitute because it prevents donor site morbidity [2]. However, both autograft and allograft have disadvantages, such as limited availability, donor site morbidity, transmission of infectious diseases, and immune-rejection reactions [2, 3]. To address these shortcomings, a new approach to bone graft has been developed.

Hydroxyapatite (HAp) and calcium phosphate (CaP) have been successfully used as bone graft materials for the regeneration of bone defects due to their physical and chemical properties and their structural similarity to natural bone. CaP-based materials have been approved by the Food and Drug Administration (FDA) for clinical applications in the fields of dental and orthopedic surgery. CaP-based materials possess bioactive, biocompatible, and osteoconductive properties [4]. Although CaP-based materials have been widely used in a clinical setting, they have no osteogenic or osteoinductive properties; however, such properties are needed for the repair and regeneration of bone defects. The osteoinductive properties of natural bone are the result of bone morphogenic proteins (BMPs) and osteogenic proteins that exist in the extracellular matrix (ECM) [5–9]. Thus, in order to produce more effective bone regeneration, CaP-based materials combined with osteoinductive materials are required.

BMPs have osteoinductive properties. Among BMP family members, BMP-2 is one of the most osteoinductive growth factors. Previous studies have demonstrated that BMP-2 can induce mesenchymal stem cells (MSCs) to differentiate into osteoblast lineages and regenerate bone [10–13]. In addition, previous studies have shown that BMP-2 stimulates osteogenic markers such as osteopontin, osteocalcin, bone sialoprotein, and alkaline phosphatase (ALP) during osteogenic differentiation* in vitro* [14, 15]. Recently, to enhance osteoblast function and bone formation, BMP-2-based delivery systems have been developed. Previous studies have reported that BMP-2 combined with collagen gels, sponges, scaffolds, hyaluronic acid, dextran, chitosan, and fibrin scaffolds induces the regeneration of bone defects [16–21].

Carboxymethyl cellulose (CMC) has natural biodegradable and biocompatible properties [22, 23] and has been used as a biomedical membrane [24, 25]. In an* in vitro *study, Leone et al. demonstrated that amidated carboxymethyl cellulose (CMCA) hydrogel is a potential filler for cartilage defects [26]. Moreover, a recent report demonstrated that a CMC/HAp hybrid hydrogel induced more osteoblast-like cell proliferation, osteogenic markers including Runx2, ALP, and collagen type I, and mineralization than did a CMC-based hydrogel without HAp [27].

On the basis of these results, we hypothesized that a CMC-based hydrogel containing BioC (biphasic calcium phosphate (BCP); tricalcium phosphate (TCP): hydroxyapatite (Hap) = 70 : 30) and BMP-2 would promote greater bone formation in a rat tibial defect model than would a CMC-based hydrogel containing BioC without BMP-2.

## 2. Materials and Methods

### 2.1. Release Kinetics of BMP-2 from CMC/BioC/BMP-2 Hybrid Materials

The cellulose material (CMC-based hydrogel containing BioC) has been integrated with two different BMP-2 solutions (0.1 mg and 0.5 mg; Cowellmedi Co., Busan, Korea) with a simple soaking manner. Released BMP-2 from CMC/BioC/BMP-2 (0.1 mg) and CMC/Bio-C/BMP-2 (0.5 mg) hybrid materials was evaluated with an enzyme-linked immunosorbent assay (ELISA) in accordance with the manufacturer's instructions by using a microplate reader (Bio-Rad, Hercules, CA, USA) at a wavelength of 450 nm. In brief, CMC/BioC/BMP-2 (0.1 mg) and CMC/BioC/BMP-2 (0.5 mg) blended materials were, respectively, soaked in a membrane bag (MWCO: 300,000), 15 mL tube with 1 mL of phosphate buffer saline (PBS) (Gibco BRL, Rockville, MD, USA). The tube was incubated at 37°C with gentle shaking at 100 rpm. At predetermined time intervals of 1, 3, 5, and 10 hr and 1, 3, 5, 7, 14, 21, and 28 days, supernatants were collected and replaced with fresh PBS for 28 days. The absorbance of the collected samples was determined with a microplate reader.

### 2.2. Animal Study

#### 2.2.1. Rat Treatments

**Figure 1:**
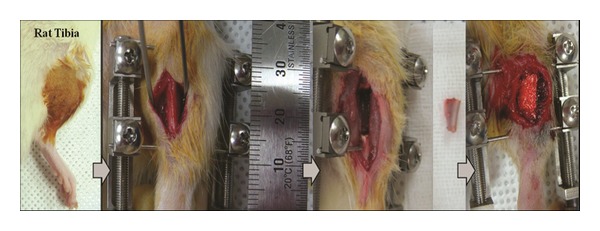
Serial photographs show the surgical procedure. After shaving, the periosteum and soft tissue were carefully retracted and two 0.9 mm K-wires (Zimmer, Warsaw, IN) were fixed to the tibia. K-wires were clamped bilaterally with the author's own-designed external fixator (U&I, Gyeonggi-do, Korea). An 8 mm defect of the right tibia was created with a cutting burr, and 150 *μ*L of CMC/BioC, CMC/BioC/BMP-2 (0.1 mg), or CMC/BioC/BMP-2 (0.5 mg) was injected into the defect, respectively.

Eight-week-old Sprague-Dawley rats (Orient Bio Co., Seongnam City, Korea) were used for the* in vivo* evaluation of CMC/BioC, CMC/BioC/BMP-2 (0.1 mg), and CMC/BioC/BMP-2 (0.5 mg) groups. The experimental protocol was approved by the Institutional Animal Care and Use Committee of the Korea University Medical Center (KUIACUC-2012-128). Experimental animals were divided into three groups: group I (*n* = 4) was implanted with CMC/BioC, group II (*n* = 4) was implanted with CMC/BioC/BMP-2 (0.1 mg), and group III (*n* = 4) was implanted with CMC/BioC/BMP-2 (0.5 mg). The rats were anesthetized with tiletamine/zolazepam (50 mg/kg; Zoletil) and xylazine (10 mg/kg; Rompun). After shaving the right tibia, the periosteum and soft tissue were carefully retracted and two 0.9 mm K-wires (Zimmer, Warsaw, IN) were fixed to the right tibia. K-wires were clamped bilaterally with the author's own-designed external fixator (U&I, Gyeonggi-do, Korea). An 8 mm defect of the right tibia was created with a cutting burr, and 150 *μ*L of CMC/BioC, CMC/BioC/BMP-2 (0.1 mg), or CMC/BioC/BMP-2 (0.5 mg) was injected into the right tibia defect, as appropriate, respectively. The subcutaneous tissue and skin were sutured with absorbable 4-0 vicryl (Ethicon, Somerville, NJ, USA) (Figure 1). The rats were allowed free movement in cages after recovery from anesthesia. One rat in group I had been dead for two days after treatment, so another rat has been treated with the same manners with others in group I.

#### 2.2.2. Bone Formation Analyses

*Plain Radiographs.* At 4 and 8 weeks after injection, samples were fixed in 3.7% paraformaldehyde solution. Radiographs of the specimens were obtained with a plain radiograph apparatus (*In Vivo* DXS 4000 Pro System, Carestream Health, Rochester, NY, USA) at 43 KVP, 2 mA, and 44 cm film-radiation beam distance for a 1.5 s exposure time.

*Microcomputed Tomography (Micro-CT) Evaluation. *At predetermined time intervals of 4 and 8 weeks, bone volume was obtained with a micro-CT system (Albira II Imaging System, Carestream Health). The CT system was operated at a voltage of 40 kV, and a current of 250 *μ*A was used with a nominal resolution of 9 *μ*m/pixel. Image analysis was performed with a bone analyzer (Molecular Imaging Analysis software; Carestream Health Inc., Woodbridge, VA, USA).

*Histological Study. *The specimens were retrieved at 4 and 8 weeks. After decalcification, the samples were embedded in paraffin. The tissues were cross-sectioned at an 8 *μ*m thickness in the longitudinal parallel direction and stained with hematoxylin and eosin (H&E) and Masson's trichrome staining. The cytoplasm of osteoblasts and bone formation were assessed with H&E staining. Mineralized bone matrix and osteoid were evaluated by Masson's trichrome staining.

### 2.3. Statistical Analysis

Data are presented as mean ± standard deviation. Statistical comparisons were carried out via one-way analysis of variance using Systat software (Chicago, IL, USA). Differences were considered statistically significant at *P < 0.05 and **P < 0.001.

## 3. Results

### 3.1. *In Vitro* BMP-2 Release Study

**Figure 2:**
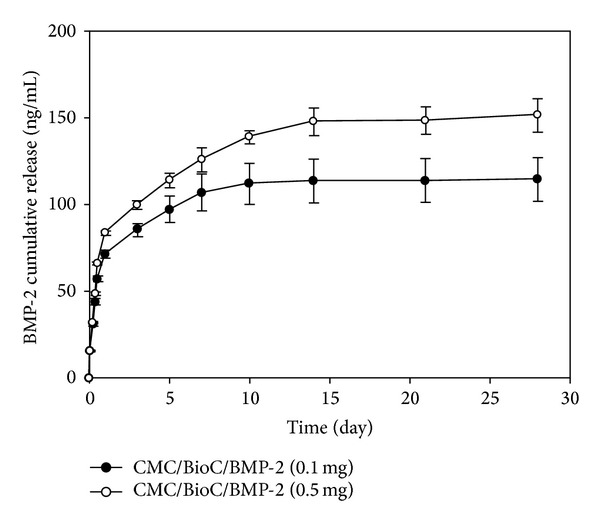
*In vitro *release profile of BMP-2 from CMC/BioC/BMP-2 hybrid materials. BMP-2 (●) from CMC/BioC/BMP-2 (0.1 mg) and BMP-2 (○) from CMC/BioC/BMP-2 (0.5 mg) hybrid materials. The error bars represent mean ± SD (*n* = 5). These experiments were repeated three times.

On the first day, the released amounts of BMP-2 were 71.55 ± 2.24 ng with CMC/BioC/BMP-2 (0.1 mg) and 83.75 ± 1.12 ng with CMC/Bio-C/BMP-2 (0.5 mg) (Figure 2). At 28 days, the released amounts of BMP-2 were 114.82 ± 12.55 ng with CMC/BioC/BMP-2 (0.1 mg) and 151.76 ± 9.57 ng with CMC/Bio-C/BMP-2 (0.5 mg).

### 3.2. Plain Radiographs and Micro-CT Analysis

**Figure 3:**
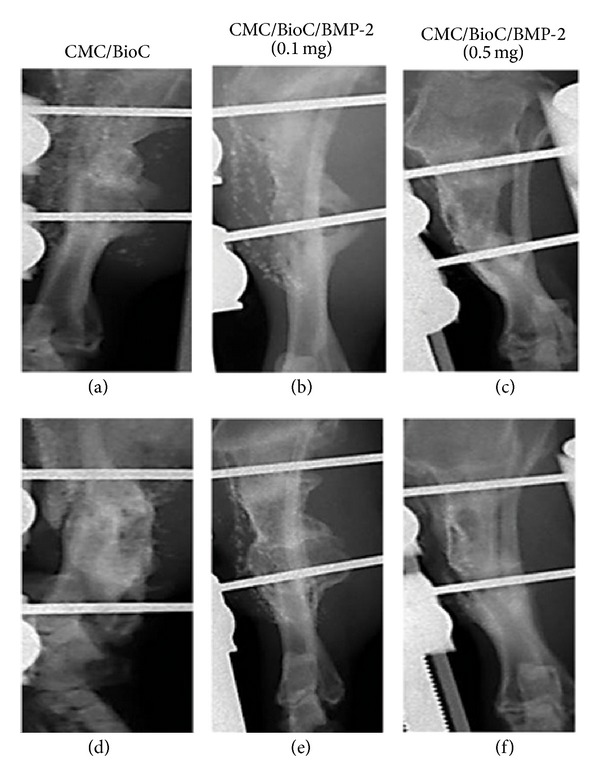
Plain radiographs of (a) CMC/BioC, (b) CMC/BioC/BMP-2 (0.1 mg), and (c) CMC/BioC/BMP-2 (0.5 mg) at 4 weeks after implantation onto the tibial defects. (d) CMC/BioC, (e) CMC/BioC/BMP-2 (0.1 mg), and (f) CMC/BioC/BMP-2 (0.5 mg) at 8 weeks after implantation onto the tibial defects. There was no mineralization at the defect site after the implantation of CMC/BioC, whereas both CMC/BioC/BMP-2 (0.1 mg) and CMC/BioC/BMP-2 (0.5 mg) experienced slight mineralization at 4 weeks. At 8 weeks after surgery, both the CMC/BioC/BMP-2 (0.1 mg) and CMC/BioC/BMP-2 (0.5 mg) groups experienced much greater mineralization than did the CMC/BioC group.

**Figure 4:**
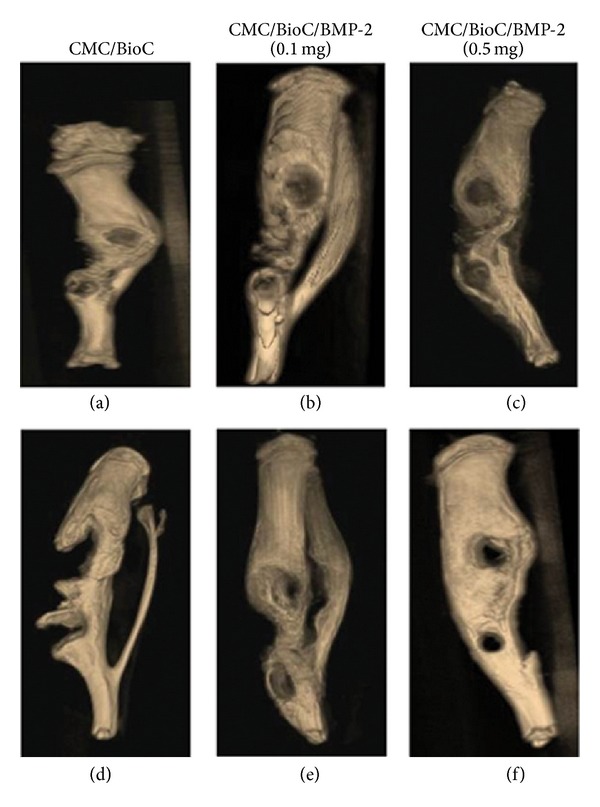
Micro-CT images of (a) CMC/BioC, (b) CMC/BioC/BMP-2 (0.1 mg), and (c) CMC/BioC/BMP-2 (0.5 mg) at 4 weeks after implantation onto the tibial defects. (d) CMC/BioC, (e) CMC/BioC/BMP-2 (0.1 mg), and (f) CMC/BioC/BMP-2 (0.5 mg) at 8 weeks after implantation onto the tibial defects. The micro-CT images revealed slight mineralization at the defect site for both the CMC/BioC/BMP-2 (0.1 mg) and CMC/BioC/BMP-2 (0.5 mg) groups but no mineralization for the CMC/BioC group at 4 weeks. At 8 weeks, mineralization was much more extensive in the CMC/BioC/BMP-2 (0.5 mg) group than in the CMC/BioC or CMC/BioC/BMP-2 (0.1 mg) group at 8 weeks.

**Figure 5:**
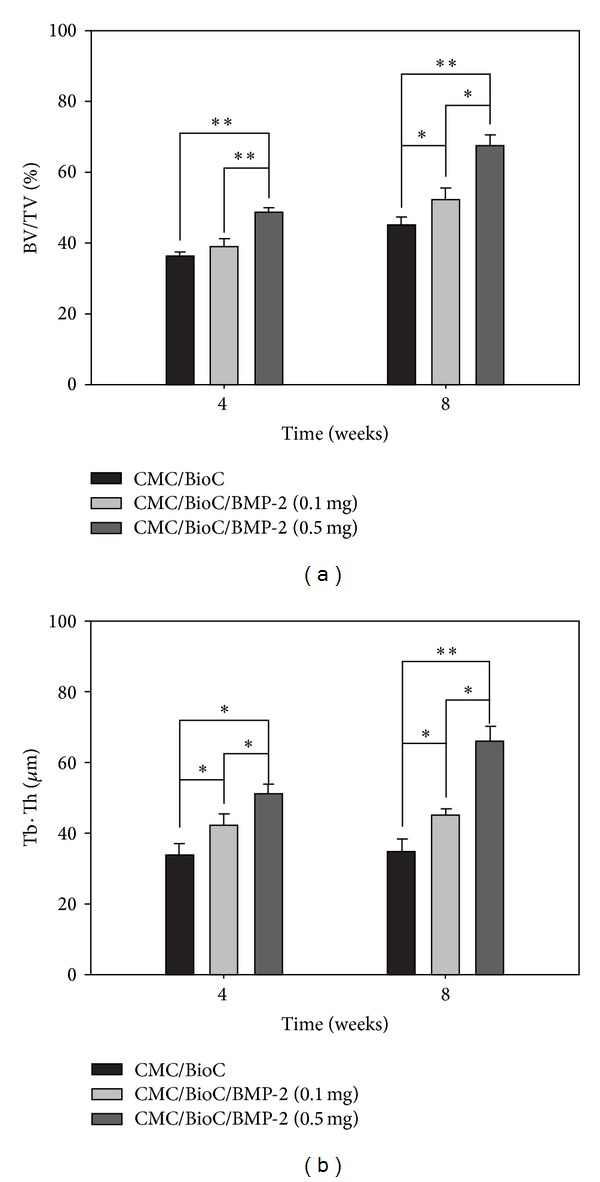
(a) Bone formation and (b) bone remodeling for the CMC/BioC, CMC/BioC/BMP-2 (0.1 mg), and CMC/BioC/BMP-2 (0.5 mg) groups at 4 and 8 weeks. The values represent mean ± standard deviation (*n* = 12) (*P < 0.05 and **P < 0.001).

At 4 weeks after surgery, the plain radiographs revealed no mineralization at the defect area for the CMC/BioC group but slight mineralization for the CMC/BioC/BMP-2 (0.1 mg) and CMC/BioC/BMP-2 (0.5 mg) groups (Figure 3). At 8 weeks after surgery, both the CMC/BioC/BMP-2 (0.1 mg) and CMC/BioC/BMP-2 (0.5 mg) groups showed much more mineralization than did the CMC/BioC group. All three groups showed much greater mineralization at 8 weeks than at 4 weeks. The micro-CT images revealed that the defect areas for both CMC/BioC/BMP-2 (0.1 mg) and CMC/BioC/BMP-2 (0.5 mg) showed slight mineralization while those for CMC/BioC did not show mineralization at 4 weeks (Figure 4). In addition, more mineralization was visible in the CMC/BioC/BMP-2 (0.5 mg) group than in the CMC/BioC or CMC/BioC/BMP-2 (0.1 mg) group at 8 weeks. Micro-CT revealed bone formation and bone remodeling. As shown in Figure 5(a), bone formation at the defect area was significantly greater for the CMC/BioC/BMP-2 (0.5 mg) group at 4 weeks than for the CMC/BioC and CMC/BioC/BMP-2 (0.1 mg) groups (**P < 0.001). A significant difference in bone formation was observed between the CMC/BioC/BMP-2 group (0.5 mg) and CMC/BioC group (**P < 0.001) and between the CMC/BioC/BMP-2 group (0.5 mg) and the CMC/BioC/BMP-2 (0.1 mg) group (*P < 0.05) at 8 weeks. At 4 weeks, there was a statistically significant difference in bone remodeling of the defect area between the CMC/BioC/BMP-2 group (0.5 mg) and the CMC/BioC group (*P < 0.05), as well as between the CMC/BioC/BMP-2 group (0.5 mg) and the CMC/BioC/BMP-2 (0.1 mg) group (*P < 0.05) (Figure 5(b)). In addition, a significantly greater amount of bone remodeling occurred in the CMC/BioC/BMP-2 (0.5 mg) group than either the CMC/BioC group (**P < 0.001) or the CMC/BioC/BMP-2 (0.1 mg) group at 8 weeks (*P < 0.05).

### 3.3. Histological Study

**Figure 6:**
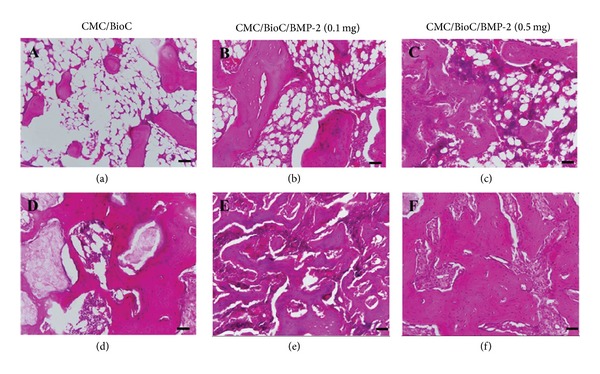
H&E staining of the rat tibial defect after implantation of (a) CMC/BioC, (b) CMC/BioC/BMP-2 (0.1 mg), and (c) CMC/BioC/BMP-2 (0.5 mg) at 4 weeks. (d) CMC/BioC, (e) CMC/BioC/BMP-2 (0.1 mg), and (f) CMC/BioC/BMP-2 (0.5 mg) at 8 weeks. The tissue of the defect area showed a much greater amount of mineralized bone tissue for the CMC/BioC/BMP-2 (0.5 mg) group than for the CMC/BioC or CMC/BioC/BMP-2 (0.1 mg) group at 4 weeks. Mineralized bone tissue was observed at the whole circumference of the defect site in the CMC/BioC/BMP-2 (0.5 mg) group but not in the CMC/BioC or CMC/BioC/BMP-2 (0.1 mg) group at 8 weeks (scale bar = 50 *μ*m).

**Figure 7:**
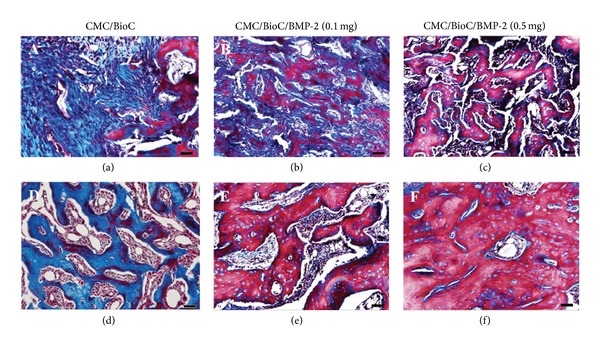
Masson's trichrome staining of the rat tibial defect after implantation of (a) CMC/BioC, (b) CMC/BioC/BMP-2 (0.1 mg), and (c) CMC/BioC/BMP-2 (0.5 mg) at 4 weeks. (d) CMC/BioC, (e) CMC/BioC/BMP-2 (0.1 mg), and (f) CMC/BioC/BMP-2 (0.5 mg) at 8 weeks. General woven bone tissue (blue color) covered the defects in the CMC/BioC group, whereas at least some mineralized bone tissue (red color) was present in the CMC/BioC/BMP-2 (0.1 mg) and CMC/BioC/BMP-2 (0.5 mg) groups at 4 weeks. At 8 weeks, much of the mineralized bone tissue on the defect site was visible in the CMC/BioC/BMP-2 (0.5 mg) group, but this amount was much lower in the CMC/BioC or CMC/BioC/BMP-2 (0.1 mg) group (scale bar = 50 *μ*m).

The H&E staining analysis revealed that the tissue of the defect area showed negligible mineralized bone tissue for the CMC/BioC group and partially mineralized bone tissue for the CMC/BioC/BMP-2 (0.1 mg) group. However, the tissue of the defect area showed a much greater amount of mineralized bone tissue for the CMC/BioC/BMP-2 (0.5 mg) group than for the CMC/BioC or CMC/BioC/BMP-2 (0.1 mg) group at 4 weeks (Figures 6(a)–6(c)). In all three groups, a much greater amount of mineralized bone tissue was observed around the defect at 8 weeks after surgery than at 4 weeks after surgery. Moreover, mineralized bone tissue was observed at the whole circumference of the defect site in the CMC/BioC/BMP-2 (0.5 mg) group but not in the CMC/BioC or CMC/BioC/BMP-2 (0.1 mg) group at 8 weeks (Figures 6(d)–6(f)). The Masson's trichrome staining analysis revealed that general woven bone tissue covered the defects in the CMC/BioC group, whereas at least some mineralized bone tissue was present in the CMC/BioC/BMP-2 (0.1 mg) and CMC/BioC/BMP-2 (0.5 mg) groups at 4 weeks (Figures 7(a)–7(c)). At 8 weeks, much of the mineralized bone tissue on the defect site was visible in the CMC/BioC/BMP-2 (0.5 mg) group, but this amount was much lower in the CMC/BioC or CMC/BioC/BMP-2 (0.1 mg) group (Figures 7(d)–7(f)).

## 4. Discussion

Osteoconductive materials, such as CaP-based materials, allow a framework for vascular invasion and cellular infiltration but do not induce mesenchymal cells to differentiate into mature bone cells. Osteoinductive materials, including growth factors such as BMPs, basic fibroblast growth factor (bFGF), platelet-derived growth factor (PDGF), and vascular endothelial growth factor (VEGF), induce new bone formation but they do not provide a framework for vascular invasion and cellular infiltration. Thus, osteoconductive materials combined with osteoinductive materials may be ideal for bone regeneration.

The objective of this study was to assess whether a CMC-based hydrogel containing BioC and BMP-2 induced a greater amount of new bone formation than did a CMC-based hydrogel containing BioC without BMP-2. The results from the plain radiographs and micro-CT demonstrated that bone formation was significantly greater in the CMC/BioC/BMP-2 (0.5 mg) group than in the CMC/BioC or CMC/BioC/BMP-2 (0.1 mg) group at 4 and 8 weeks. Thus, these findings demonstrate that CMC/BioC/BMP-2 (0.5 mg) induces rapid bone formation at an earlier stage than does CMC/BioC or CMC/BioC/BMP-2 (0.1 mg). As expected from the plain radiographs and micro-CT data, the histological study data revealed that the CMC/BioC/BMP-2 (0.5 mg) group had more mineralized bone tissue at the defect site than did the CMC/BioC and CMC/BioC/BMP-2 (0.1 mg) groups at 4 weeks. In all groups, a greater amount of mineralized bone tissue was observed at the defect site at 8 weeks than at 4 weeks. The entire defect area was covered with mineralized bone tissue at 8 weeks in the CMC/BioC/BMP-2 (0.5 mg) group but not in the CMC/BioC or CMC/BioC/BMP-2 (0.1 mg) group.

These results are consistent with those of previous studies. Lin et al. [28] reported that BMP-2-immobilized heparin-bound demineralized bone matrix (HC-DBM) showed higher alkaline phosphatase (ALP) activity (2 weeks), more calcium deposition (4 and 8 weeks), and more bone formation than that of controls after subcutaneous implantation in rats. Zhao et al. [29] demonstrated that a BMP-2-absorbed monoclonal antibodies conjugated DBM (MAbs-DBM) group experienced greater osteogenic differentiation in an* in vitro *study and greater ectopic bone formation in an* in vivo* study than the control group. In a previous study, we found that woven bone covered the whole circumference more often in a BMP-2-coated tricalcium phosphate/hydroxyapatite group than in a tricalcium phosphate/hydroxyapatite group in a rat model of femoral distraction osteogenesis [30, 31]. Kim et al. [32] and Park et al. [33] reported that BMP-2-coated biphasic calcium phosphate (BCP) granules or blocks supported significantly greater bone formation than BCP granules or blocks in a rat model of calvarial defects. Moreover, Choi et al. [34] showed that implantation of BMP-2/BCP granules onto 6 mm diameter defects of the maxillary sinus of rabbits led to enhanced bone formation compared with the control group. Finally, a recent report demonstrated that new bone formation at a bone defect in the middle ear after mastoid surgery was greater in the presence of BMP-2/BCP scaffolds [35].

Our results suggest that CMC/BioC/BMP-2 hybrid materials induce greater bone formation at an earlier stage through release of BMP-2 than CMC/BioC hybrid materials. However, our* in vivo* study has some limitations. For example, long-term evaluation greater than 8 weeks is needed to compare the quality and architecture of new bone formation between control and implanted groups. More comprehensive analyses that include the histology and angiogenesis of new bone and biomechanical testing are needed. Finally, the effects of long-term release of BMP-2 require further investigation.

In conclusion, CMC/BioC/BMP-2 (0.5 mg) hybrid materials implanted in a rat tibial defect model led to greater bone formation than did CMC/BioC and CMC/BioC/BMP-2 (0.1 mg) hybrid materials. Thus, CMC/BioC/BMP-2 hybrid materials may be useful in an injectable substrate for the clinical application of the regeneration of bone defects in the orthopedic field.
